# Genome-wide analysis of TMD-associated local ribosome accumulation reveals stress- and eS7-associated remodeling in *Saccharomyces cerevisiae*

**DOI:** 10.1093/nargab/lqag137

**Published:** 2026-11-10

**Authors:** Ming Li

**Affiliations:** Department of Chemistry, Graduate School of Science, Kyoto University, Kitashirakawa Oiwakecho, Sakyo-ku, Kyoto 606-8502, Japan

## Abstract

Local ribosome accumulation near transmembrane domains (TMDs) may report cotranslational events in membrane-protein biogenesis, but its spatial organization and stress responsiveness remain unclear. *Saccharomyces cerevisiae* ribosome-profiling data were reanalyzed using a replicate-aware, TMD-centered workflow. Two untreated wild-type baseline samples showed maximal accumulation approximately 75 nucleotides downstream of annotated TMD starts. At this position, 2896 TMDs met strict eight-sample coverage criteria; 2892 were testable using quasi-likelihood negative-binomial models of target-window counts relative to gene-wide non-TMD coding background. Forty-one TMDs showed an ER-stress effect in wild-type cells, six in eS7-4KR cells, and 22 showed a genotype-by-stress interaction at false discovery rate (FDR) < 0.05. Interaction-effect directions were concordant across models, but only eight of the 22 primary interactions met FDR < 0.05 in both target/background models; 16 remained significant after global correction across 11 positions. Baseline signals were heterogeneous. Aromatic-residue fraction remained associated with local accumulation in a prespecified joint model. Mean-based upper-tail topology associations weakened with one-TMD-per-protein resampling; continuous protein-level comparisons were nonsignificant. Functional enrichment was sensitive to the number of tested TMDs per gene and was treated as exploratory. These results identify spatially resolved, stress- and eS7-associated ribosome-occupancy differences without equating footprint enrichment with proven translational pausing.

## Introduction

Membrane proteins perform essential roles in transport, signaling, membrane trafficking, and cellular homeostasis [1]. Their biogenesis requires coordination between polypeptide synthesis, targeting, membrane insertion, translocation, folding, and quality control [2–5]. Transmembrane domains (TMDs) are consequently translated in a context in which ribosome position and kinetics may be coupled to the signal-recognition particle, translocon, and membrane-insertion machinery.

Ribosome profiling provides nucleotide-resolution maps of ribosome-protected fragments and has been widely used to investigate translational regulation [6–8]. Ribosome collisions and stalled-ribosome ubiquitination link elongation perturbation to quality control [9, 10], but footprint enrichment alone does not establish either process. This distinction is particularly important near TMDs because cotranslational targeting or insertion may occur after the nascent segment has advanced beyond its annotated boundary.

Endoplasmic reticulum (ER) stress changes protein homeostasis, translation, membrane trafficking, and quality-control pathways [11–13]. Matsuki and colleagues generated yeast ribosome-profiling data after tunicamycin treatment and showed that ubiquitination of ribosomal protein eS7 contributes to selective translational responses and stress outcome [14]. Their matched biological replicates for wild-type and ubiquitination-deficient eS7-4KR cells permit a direct test of whether stress-associated local ribosome occupancy differs between genotypes.

This study systematically evaluates TMD-centered ribosome occupancy by scanning signals relative to TMD starts, retaining biological replicates in count models, testing genotype dependence through a direct genotype-by-stress interaction, and using the actual analyzable universe for topology, sequence, overlap, and functional analyses. The resulting workflow is configurable for external BAM, annotation, TMD, and sample-metadata inputs. It identifies a reproducible downstream signal and a restricted set of stress- and eS7-associated events while preserving the distinction between ribosome accumulation and mechanistically demonstrated pausing.

## Materials and methods

### Ribo-seq datasets and experimental design

Ribosome-profiling datasets were obtained from GEO accession GSE128578 [14]. Baseline positional and structural analyses used untreated wild-type samples SRR8756003 and SRR8756004. Replicate-aware stress analyses used eS7-WT control (SRR8756008 and SRR8756009), eS7-WT after 4 h tunicamycin treatment (SRR8756010 and SRR8756011), eS7-4KR control (SRR8756012 and SRR8756013), and eS7-4KR after 4 h treatment (SRR8756014 and SRR8756015). Biological replicates were retained as separate samples.

### Read processing, alignment, and quality control

Reads were aligned to the *S. cerevisiae* R64-1-1 reference genome with Bowtie2 v2.5.5 [15], and alignment files were processed with SAMtools v1.23.1 [16]. Only primary mapped alignments with footprint lengths of 26–32 nt were used. The paper configuration used a minimum mapping-quality threshold of 0 and retained duplicate-flagged alignments; unmapped, secondary, and supplementary alignments were excluded. Quality-control outputs included exact mapping statistics, footprint-length distributions, coding-sequence frame periodicity, read-length-dependent P-site offsets, assignment summaries, and replicate correlations. Mapping percentages ranged from 92.6% to 97.2% (Supplementary Fig. S1 and Supplementary Table S2).

### P-site calibration and assignment

P-site calibration evaluated candidate offsets from 10 to 17 nt independently for each sample and read length. At most the first 3 000 000 BAM alignment records per sample were examined during offset calibration and CDS-start metagene quality control, with the alignment-eligibility filters described above applied within this cap. Frame-0 fractions were averaged across phase-equivalent candidate offsets; the phase with the highest coding-sequence periodicity was selected and mapped to a canonical output offset of 12, 13, or 14 nt. Neither TMD signals nor differential-model results were used for calibration. CDS-start metagene profiles were generated separately after P-site assignment as a quality-control diagnostic and were not used to select offsets. Strand-aware P-site coordinates were computed from the alignment 5′ end after accounting for read orientation. Because some sample-specific choices were correlated with genotype, an additional common-offset sensitivity analysis derived one read-length-specific consensus phase by first averaging phase-equivalent candidates within each library and read length and then giving equal weight to each of the eight modeled libraries. The selected consensus phase was likewise mapped to a canonical 12-, 13-, or 14-nt output offset. The primary interaction model was then repeated with these common offsets. Effect comparisons used the 2892 TMDs testable under both calibrations. Benjamini–Hochberg correction for the common-offset model was performed across its complete 2895-TMD test universe, rather than within the shared set or the 22 primary interactions. The complete offset tables, periodicity diagnostics, and primary-versus-common-offset comparison are provided in Supplementary Figs S1 and S8 and Supplementary Tables S2 and S4.

### TMD annotation and coordinate convention

Membrane-protein and TMD annotations were obtained from UniProt and mapped to the R64-1-1 genome annotation [17]. The annotation comprised 5028 TMDs in 997 genes (Supplementary Table S1). Protein coordinates were converted to 1-based inclusive CDS-relative nucleotide intervals. GTF-derived genomic positions were converted to 0-based coordinates before comparison with 0-based BAM/P-site coordinates. All conversions were strand aware and were validated against CDS boundaries. Target windows extending beyond a coding sequence or overlapping another annotated TMD were flagged and excluded from primary inference.

### Spatial profiles and the primary target window

For each sample and TMD, sense-strand P-site counts were quantified in 31-nt windows centered from −30 to +120 nt in 15-nt increments relative to the annotated TMD start. For the two untreated wild-type baseline samples, each eligible TMD was first normalized to its gene-wide non-TMD coding background and then contributed equal weight to the metagene. The across-replicate mean was maximal at +75 nt and remained elevated at +90 nt. A 31-nt window centered at +75 nt (+60 to +90 nt) was therefore specified as the primary target window before fitting the eight-sample genotype-by-stress model. Window selection did not use interaction significance.

For descriptive baseline analysis, local accumulation was calculated as:


\begin{eqnarray*}
{\mathrm{Local}}\,{\mathrm{signal}} &=& \log _{ 2}\big[\big(\big({\mathrm{target}}\,{\mathrm{P-sites}} + 0.5)/31\big)\\&&/\big(\big({\mathrm{background}}\,{\mathrm{P-sites}} + 0.5\big)\\&&/{\mathrm{background}}\,{\mathrm{length}}\big)\big].
\end{eqnarray*}

The gene-wide background comprised translated coding positions outside all annotated TMD-associated intervals. For every scanned position, the complete 31-nt target window for that TMD was additionally excluded from its own background, including any target bases outside the transcript-level TMD exclusion mask. This strategy avoids target/background overlap as well as local background windows that cross CDS boundaries or neighboring TMDs. Local footprint enrichment is reported as ribosome accumulation or occupancy, not as proof of a discrete translational pause.

### Coverage eligibility and analysis populations

Coverage status was evaluated for every TMD–sample combination. Primary models required a structurally valid target window and at least 20 background P-sites in every one of the eight modeled samples. This strict intersection contained 2896 TMDs. Four TMDs had zero target counts across all eight samples and were not estimable in the count models, leaving 2892 statistically tested TMDs. Per-sample, design-cell, comparison-specific, and strict-intersection counts are reported in Supplementary Fig. S4 and Supplementary Table S2.

### Replicate-aware differential analysis

Differential local accumulation was tested with edgeR quasi-likelihood negative-binomial models [18–20]. Target-window P-site counts were the response. The expected exposure for each TMD and sample was (background P-sites +0.5) × target-window length/background length; its logarithm was supplied as an observation-specific offset. The design was ~ genotype * stress, with WT and 4KR genotype levels and control and tunicamycin stress levels. Quasi-likelihood tests assessed the WT stress effect, the 4KR stress effect, and the genotype-by-stress interaction. Benjamini–Hochberg FDRs were calculated separately for each contrast, and FDR < 0.05 defined statistical evidence. Previously used absolute-change classifications were retained only as historical descriptive outputs and were not used for inference.

### Alternative target/background sensitivity model

To examine whether treating background-derived exposure as fixed underestimated background uncertainty, an alternative edgeR feature-usage model was fitted to target and background counts from the same eight libraries. For each TMD, target-window and gene-wide non-TMD background counts were represented as two feature rows. This feature-usage formulation treats the target and background as paired features nested within each TMD, analogous to differential exon-usage testing, so each contrast tests whether the condition-dependent change differs between the target window and its within-gene background. A DGEList was normalized with normLibSizes; dispersions were estimated with estimateDisp(…, robust = TRUE) under the sample-level design ~ genotype * stress; and quasi-likelihood fits were obtained with glmQLFit(…, robust = TRUE). WT stress, 4KR stress, and interaction contrasts were constructed from the four design-cell means, with the interaction defined as (4KR stress − 4KR control) − (WT stress − WT control). For each contrast, diffSpliceDGE(…, geneid = TMD, exonid = region, robust = TRUE) compared the target-feature coefficient with the background-feature coefficient within each TMD, and topSpliceDGE(test = “Simes”, number = Inf) supplied TMD-level Simes *P-*values and Benjamini–Hochberg FDRs. Region length affects the within-TMD baseline difference but cancels from these between-condition target-versus-background contrasts; no fixed background-derived exposure was used in this sensitivity model. This model was prespecified as a sensitivity analysis of background-offset uncertainty, not as a second primary event definition or independent validation. Primary and sensitivity estimates were compared for direction, rank correlation, FDR, and overlap of significant sets.

### Replicate and positional sensitivity

Within-condition consistency was summarized using Pearson correlations over TMDs passing the configured coverage threshold in both replicates. The four correlations were 0.672 for WT control, 0.629 for WT stress, 0.669 for 4KR control, and 0.600 for 4KR stress (Supplementary Fig. S2). The complete model was repeated at all 11 positions. In addition to center-wise correction, Benjamini–Hochberg correction was applied separately for each contrast across the complete family of 32 723 tested TMD-by-position hypotheses. Eligibility was evaluated separately at each scanned center because target-window structural validity, background coverage after target exclusion, and all-zero target filtering were position specific. The number of tested TMDs therefore ranged from 1623 to 3895 across centers, and 32 723 represents the sum of the 11 position-specific test sets. The +75-nt analysis remained the prespecified primary result.

### Protein-aware topology analysis

Topology was analyzed at the protein level so that multi-pass proteins did not contribute multiple independent proteins. For each gene, mean, median, maximum, and high-signal fractions were summarized across eligible TMDs. The primary continuous comparison used protein-mean signal and a two-sided Mann–Whitney test. Proteins in the highest decile were classified as high-signal, category percentages and a two-sided Fisher exact test were reported, and a complementary TMD-level model used a single-pass indicator and log_2_ background coverage with gene-clustered standard errors. To assess dependence on the decile cutoff, the Fisher analysis was repeated for the highest 5%, 10%, 15%, and 20% of protein-mean signals, with odds ratios and 95% confidence intervals reported. *P-*values from these four cutoff-sensitivity tests were nominal and were not adjusted for testing multiple cutoffs.

To assess sensitivity to unequal TMD averaging, one eligible TMD was sampled uniformly per protein in 10 000 iterations. The same 615 proteins (217 single-pass and 398 multi-pass), annotation-based topology labels, and 2214 frozen baseline TMD scores were retained. The original upper 5%, 10%, 15%, and 20% definitions were applied to the pooled 615 sampled scores in each iteration, using linear quantile interpolation and including scores at or above each threshold. Two-sided Fisher tests compared single-pass and multi-pass tail membership. Odds ratios are summarized by their medians and 2.5%–97.5% resampling ranges. These ranges describe TMD-selection variability conditional on the observed data, not confidence intervals. Code, fixed parameters, and reproducibility checks are supplied in the R2 source-code addendum.

### Sequence and flanking-region features

Features included TMD length, hydrophobic, aromatic, bulky, charged, and proline/glycine fractions, GRAVY score, and charge-related features in 10-residue N- and C-terminal flanks. Marginal linear models related each standardized feature to baseline +75-nt signal while adjusting for topology and background coverage, with gene-clustered standard errors. Joint-model terms were specified without reference to their univariate significance: GRAVY represented overall hydropathy; aromatic, proline/glycine, and charged fractions represented nonredundant composition classes; TMD length and C-flank net charge represented structural and flank properties; and topology and background coverage were covariates. Bulky and aggregate hydrophobic fractions were omitted from the joint model because of overlap with GRAVY and aromatic composition, rather than because of their observed *P-*values. Variance-inflation factors were calculated for all joint predictors, and sequence-term *P-*values were FDR corrected.

To characterize the TMDs identified by the three differential contrasts, response-set membership was defined directly from the frozen primary model at Benjamini–Hochberg FDR < 0.05: WT stress-responsive, 4KR stress-responsive, and genotype-by-stress interaction TMDs. Sequence and topology annotations were extracted independently of baseline-signal eligibility for all 2892 statistically testable TMDs. Six prespecified nonredundant features—TMD length, GRAVY, aromatic fraction, proline/glycine fraction, charged fraction, and C-flank net charge—were tested in separate binomial models of response-set membership, with single-pass topology and log_2_ mean background coverage as covariates and gene-clustered robust standard errors. Benjamini–Hochberg correction was applied globally across the 18 set-by-feature tests; within-set FDRs were also tabulated descriptively. Because the 4KR set contained only six TMDs, the corresponding analysis was treated as descriptive owing to limited statistical power. Topology was analyzed at the protein level: A gene was selected when at least one of its TMDs belonged to the relevant response set, and single-pass versus multi-pass membership was tested with a two-sided exact test that conditioned on the number of selected genes within each distinct tested-TMD-count stratum. The three topology *P-*values were Benjamini–Hochberg corrected. These *post hoc* characterization analyses did not redefine the primary differential sets.

### Dataset overlap analysis

Basal high-signal, WT stress-responsive, 4KR stress-responsive, and genotype-by-stress interaction sets were compared using pair-specific eligibility rules. Basal high-signal TMDs were defined by the highest decile of baseline signal. Comparisons involving the basal set were restricted to the baseline-eligible intersection, comprising 2208 TMDs and 614 genes. Comparisons among differential sets only used the complete primary-tested universe of 2892 TMDs and 772 genes. Overlap counts, fractions, Jaccard indices, and two-sided Fisher exact tests were calculated at both TMD and gene levels. Benjamini–Hochberg correction was applied across the six pairwise tests separately at TMD and gene levels.

### Gene ontology analysis

GO enrichment used the 772 membrane-protein genes that were testable in the direct interaction model as the universe. Because genes with more analyzed TMDs have more opportunities to contain a significant event, every GO term was evaluated three ways: an unadjusted Fisher test; a logistic model containing term membership and log-transformed tested-TMD count; and an exact opportunity-stratified test. Each distinct integer value of tested TMDs per gene defined one stratum. Within stratum s, the population size (N_s_), number of selected genes (n_s_), and number of term-annotated genes (K_s_) were fixed, so the number of selected term genes followed a hypergeometric distribution. Convolution of these stratum-specific distributions gave the null distribution for the total overlap, and the reported exact *P-*value was its upper-tail probability at or above the observed total. Term mappings were restricted to the 772-gene universe, and 1502 terms represented by at least three genes were tested. Benjamini–Hochberg correction was applied separately to each approach. Interpretation was based primarily on the exact opportunity-stratified result; Fisher and logistic results were retained to expose sensitivity to structural opportunity and small-sample modeling.

### Reusable workflow and reproducibility

The analysis was generalized as a configuration-driven TMD-centered Ribo-seq workflow (tmdriboseq v1.0.0). Inputs comprise BAM/BAI files, GTF/GFF annotation, a documented TMD table, sample metadata, and YAML configuration. Modules generate QC, P-site offsets, spatial profiles, coverage-flow tables, replicate-aware differential tests, position-wide multiplicity correction, topology, sequence, overlap, and enrichment outputs. A paper configuration reproduces the core analyses, whereas an example configuration documents use with external datasets. The one-TMD-per-protein sensitivity analysis was run separately on the frozen baseline scores using the accompanying R2 source-code addendum. The audited run used Python 3.13.9, NumPy 2.3.5, pandas 2.3.3, pysam 0.24.0, SciPy 1.16.3, statsmodels 0.14.5, R 4.6.0, and edgeR 4.10.3. Exact configuration and input checksums, runtime versions, stage summaries, and Git provenance are recorded in the machine-readable run manifest. Pinned Python dependencies, the Conda environment, and a 26-test automated suite are supplied; all 26 tests pass in the complete paper-reproduction environment, whereas a source-only checkout passes 25 tests and skips the corrected-paper regression test until the reproduction outputs have been generated. The analysis structure is summarized in Fig. 1. The workflow does not automatically infer biological pausing or replace organism-specific annotation review.

**Figure 1.**
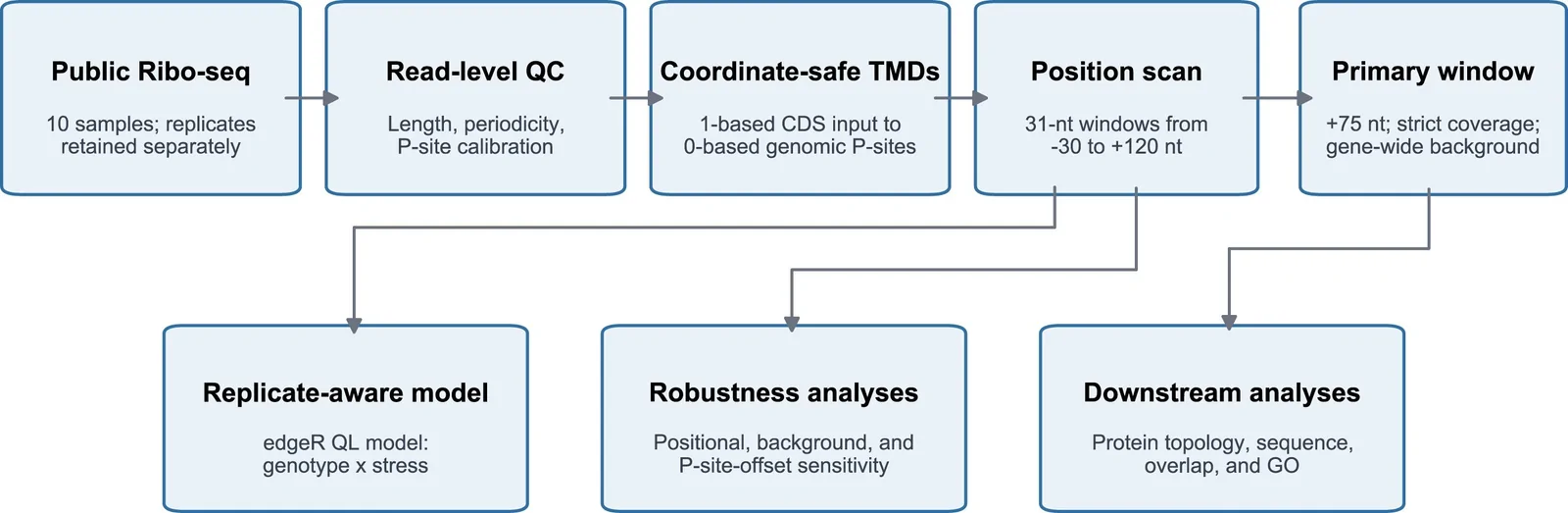
Reusable, coordinate-safe workflow for TMD-associated local ribosome accumulation. Public Ribo-seq data are processed through read-level QC, independently calibrated P-sites, coordinate-safe TMD mapping, spatial scanning, strict coverage eligibility, replicate-aware differential models, sensitivity analyses, and protein-aware downstream analyses.

## Results

### Quality control identifies a downstream TMD-associated signal

The annotation contained 5028 TMDs in 997 membrane-protein genes. Ten Ribo-seq samples showed mapping percentages of 92.6%–97.2%, canonical footprint-length enrichment, and read-length-dependent periodicity supporting sample-specific P-site offsets (Supplementary Fig. S1 and Supplementary Table S2).

The equally weighted baseline metagene did not show a dominant peak at the annotated TMD start. Both untreated wild-type replicates instead showed a broad downstream increase, and the across-replicate mean of the scanned 31-nt windows was maximal at +75 nt (Fig. 2). This position is compatible with processes associated with nascent TMD emergence, targeting, or insertion, but the analysis does not distinguish among those possibilities. Subsequent results therefore refer to TMD-associated local ribosome accumulation.

**Figure 2.**
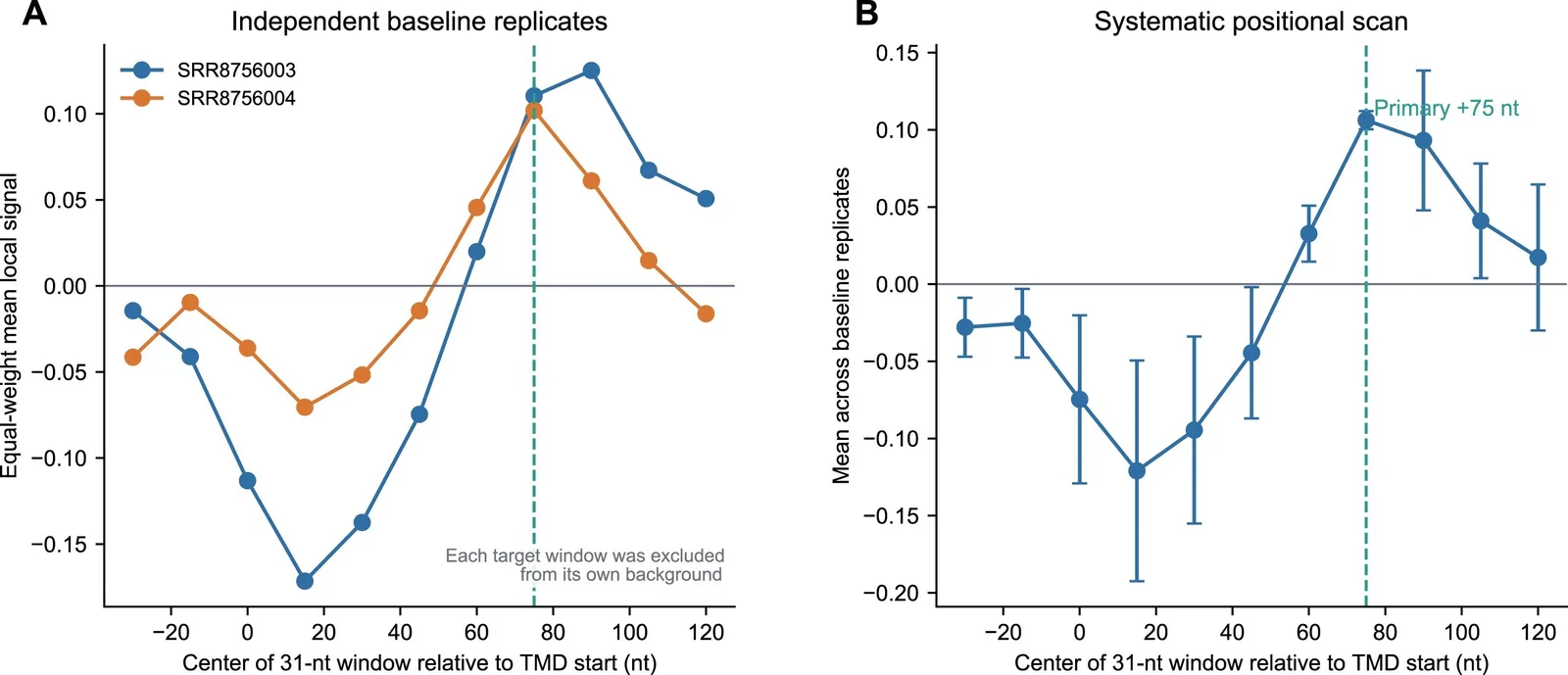
Local ribosome accumulation is maximal downstream of annotated TMD starts. (**A**) Equal-weight metagene profiles for independent untreated wild-type replicates. Each eligible TMD was normalized before averaging, and each scanned target window was excluded completely from its own background. (**B**) Across-replicate mean signal for 31-nt windows centered from −30 to +120 nt. Points show the mean across the two baseline replicate-specific means, and error bars show their standard deviation. The dashed vertical line marks the +75-nt primary-window center.

### Baseline accumulation is heterogeneous and sequence-associated

Strict baseline criteria retained 2214 TMDs in 615 proteins. Mean +75-nt signals ranged from −3.71 to 3.53 (median 0.157; mean 0.113), with a distinct high-signal tail that included TMDs from *HXT6, HXT7, AUS1, ITR1*, and other membrane proteins (Fig. 3A and B and Supplementary Table S3).

**Figure 3.**
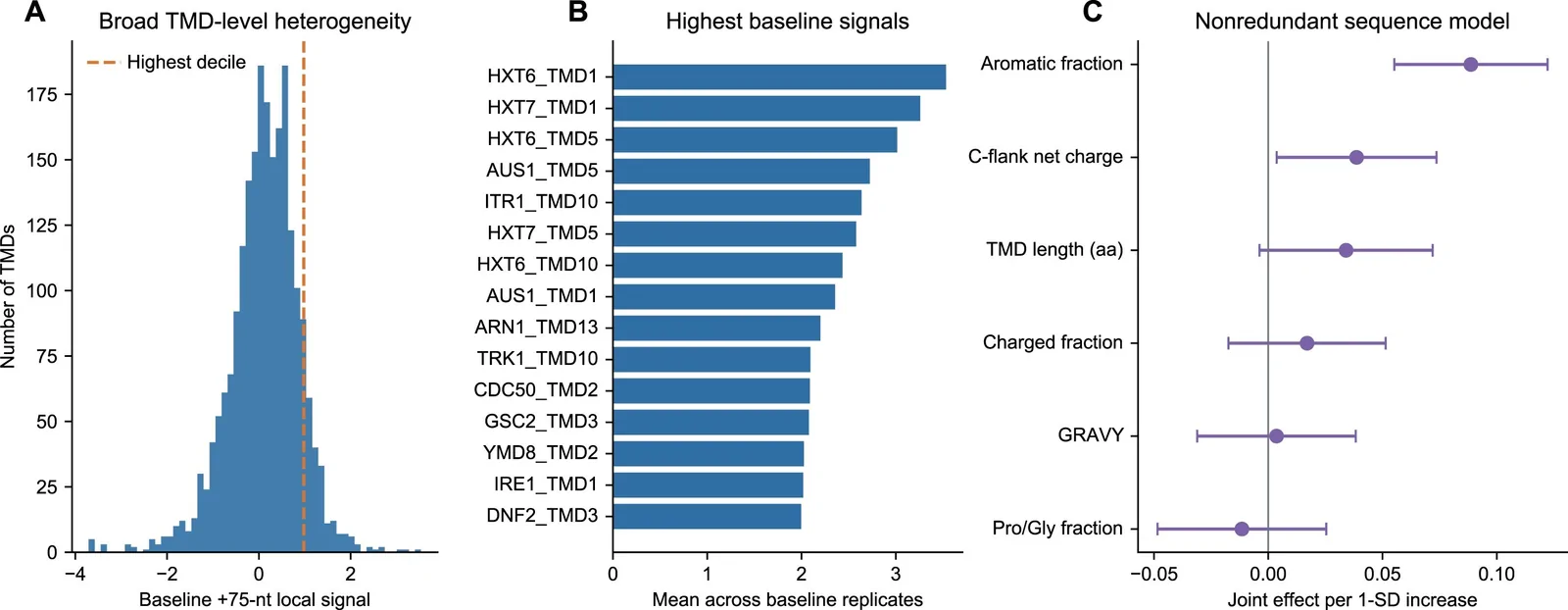
TMD-associated local accumulation is heterogeneous and sequence-associated. (**A**) Distribution of baseline +75-nt signals among 2214 TMDs; the dashed line marks the highest decile. (**B**) Fifteen highest-scoring TMDs. (**C**) Standardized coefficients and 95% confidence intervals from the nonredundant joint sequence model with gene-clustered standard errors.

Marginal models identified positive associations for aromatic and bulky residue fractions after correction, whereas several hydrophobicity, flank-charge, and proline/glycine effects were weaker (Supplementary Fig. S6 and Supplementary Table S3). In the nonredundant joint model, aromatic fraction was the only configured sequence term that remained significant after FDR correction (β = 0.0887 per SD, FDR = 1.32 × 10^−6^; Fig. 3C). GRAVY was not associated after inclusion in the joint model (β = 0.0036, FDR = 0.838), and the maximum variance-inflation factor was 1.47. C-flank net charge was nominally associated but did not pass FDR correction (β = 0.0386, FDR = 0.0907). These are associations rather than causal sequence determinants.

### Upper-tail topology associations are sensitive to TMD aggregation

Protein-level analysis included 217 single-pass and 398 multi-pass proteins. Thirty-eight single-pass proteins (17.5%) and 24 multi-pass proteins (6.0%) fell in the highest decile of protein-mean signal, corresponding to an odds ratio of 3.31 (Fisher *P* = 1.70 × 10^−5^; Fig. 4A). Using protein-mean signals, odds ratios were 3.57, 3.31, 2.60, and 2.51 for the highest 5%, 10%, 15%, and 20%, respectively, with all nominal, unadjusted Fisher *P-*values < .001 (Fig. 4C and Supplementary Table S3). In contrast, the continuous protein-level distributions did not differ (Mann–Whitney *P* = .565), and the clustered TMD-level topology coefficient was not significant (*P* = .584; Fig. 4B). When one TMD was sampled per protein, the median odds ratios for the same upper 5%, 10%, 15%, and 20% definitions were 0.87, 1.09, 1.07, and 1.17, respectively (2.5%–97.5% resampling ranges: 0.62–1.35, 0.67–1.27, 0.81–1.26, and 0.86–1.39; Fig. 4D and Supplementary Table S3). The attenuation indicates that the original mean-based upper-tail association is sensitive to the number of TMDs contributing to each protein mean. Accordingly, this association is no longer interpreted as evidence for biological enrichment of single-pass proteins in the high-signal tail.

**Figure 4.**
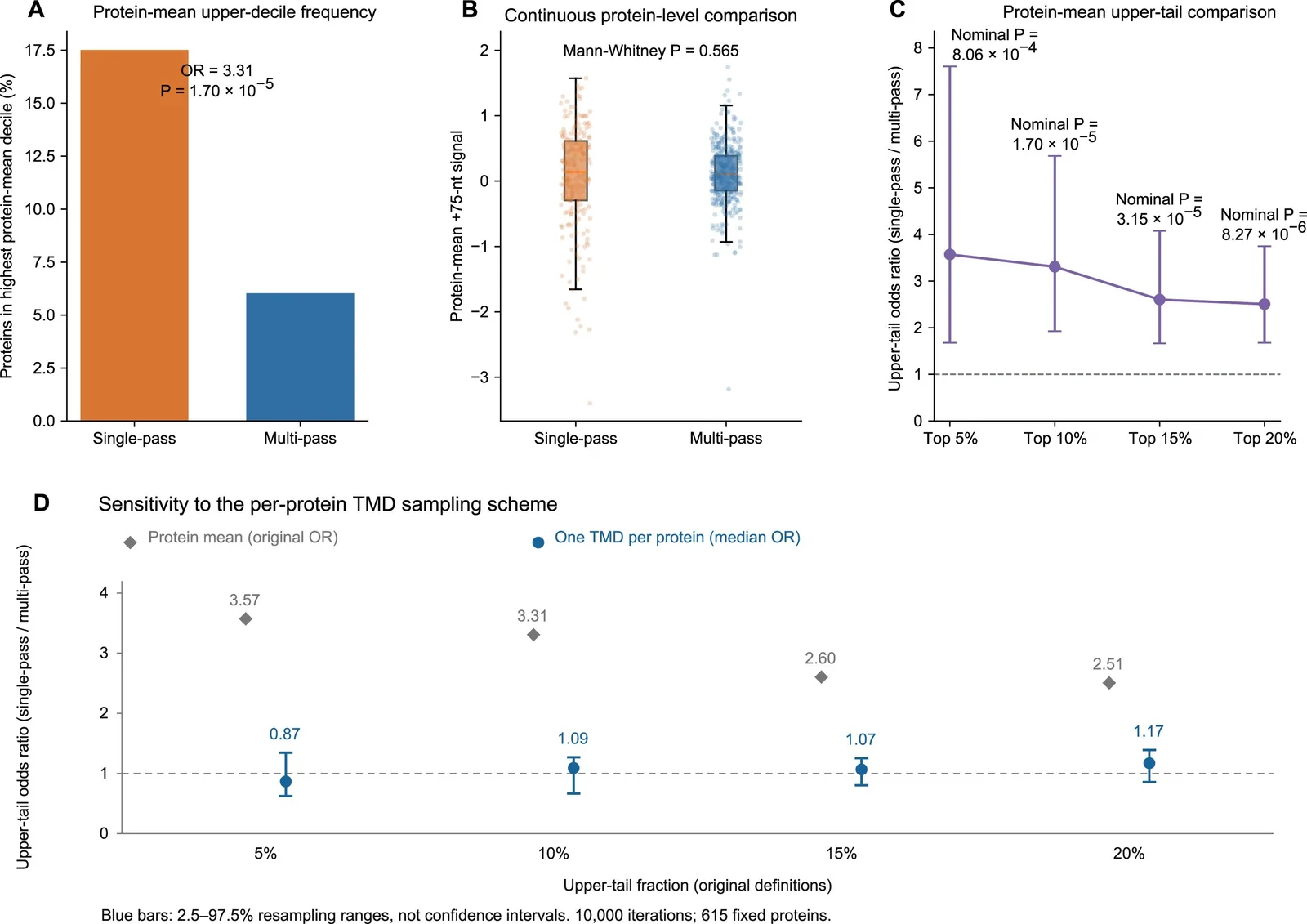
Topology comparisons using protein means and one TMD per protein. (**A**) Percentages of single-pass and multi-pass proteins in the highest decile of protein-mean signals. (**B**) Continuous protein-mean distributions shown as boxplots with all protein-level observations overlaid. (**C**) Odds ratios and 95% confidence intervals comparing single-pass and multi-pass membership in the highest 5%, 10%, 15%, and 20% of protein-mean signals; displayed Fisher *P-*values are nominal and unadjusted. (**D**) Original protein-mean odds ratios (diamond markers) compared with median odds ratios from 10 000 one-TMD-per-protein iterations (circle markers) at the same four upper-tail definitions. Error bars show the 2.5%–97.5% resampling ranges, not confidence intervals; the dashed line marks an odds ratio of one.

### ER stress alters a selective subset of TMD-associated signals

Of 2896 TMDs in the strict eight-sample coverage intersection, 2892 were statistically testable. The replicate-aware model identified 41 WT stress-responsive TMDs and six 4KR stress-responsive TMDs at FDR < 0.05 (Fig. 5A and B and Supplementary Table S4). These counts are substantially smaller than those obtained from threshold-based classifications that did not model biological variability.

**Figure 5.**
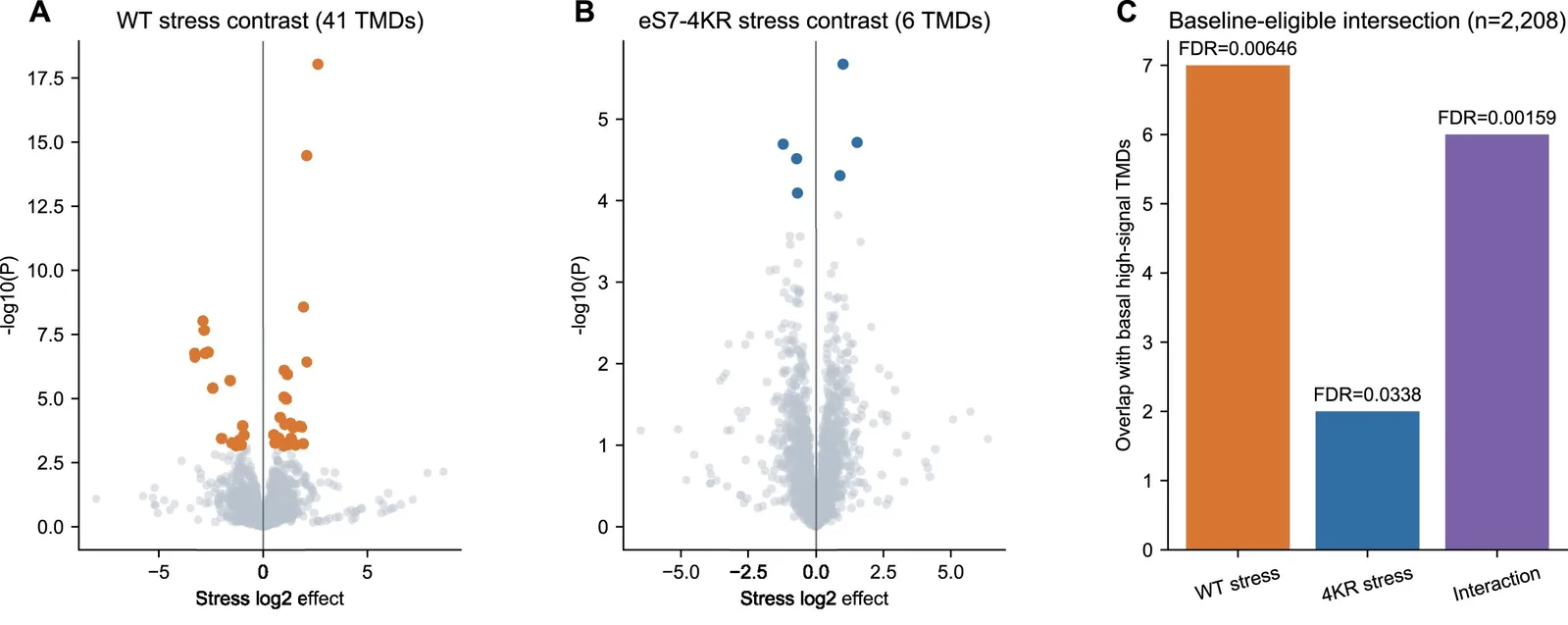
ER stress alters local ribosome accumulation at selective TMDs. (**A**) Volcano plot for the WT stress contrast; points with FDR < 0.05 are highlighted. (**B**) Volcano plot for the eS7-4KR stress contrast; points with FDR < 0.05 are highlighted. (**C**) Numbers of TMDs shared between the basal highest-decile set and the WT stress, eS7-4KR stress, and genotype-by-stress interaction sets within the 2208-TMD baseline-eligible intersection; labels show FDRs from the corresponding overlap tests.

Of the 41 WT stress-responsive TMDs, 22 were included in the 2208-TMD baseline-eligible intersection; the corresponding numbers were three of six for the 4KR stress set and 12 of 22 for the genotype-by-stress interaction set. Within this intersection, the basal high-signal set overlapped seven of 22 WT stress-responsive TMDs (odds ratio = 4.28, Fisher *P* = .00431, FDR = 0.00646), two of three 4KR stress-responsive TMDs (odds ratio = 18.05, *P* = .0282, FDR = 0.03384), and six of 12 genotype-by-stress interaction TMDs (odds ratio = 9.17, *P* = .000531, FDR = 0.00159; Fig. 5C). The small 4KR set within this eligible intersection limits the precision of its odds ratio. Differential-only comparisons used the complete 2892-TMD primary universe: 16 of 41 WT stress-responsive TMDs overlapped the 22 interactions (odds ratio = 303, FDR = 4.00 × 10^−26^), whereas two of six 4KR stress-responsive TMDs overlapped the interaction set (odds ratio = 71.65, FDR = 0.00163; Supplementary Table S5). These results distinguish baseline accumulation, within-genotype stress response, and differential genotype response while showing that they are related.

### Direct interaction modeling identifies 22 eS7-associated differences

The direct genotype-by-stress model identified 22 TMDs in 14 genes at FDR < 0.05 (Fig. 6A and B and Supplementary Table S4). The genes were *ATO3, ATX2, FLC2, FMP42, HXT1, HXT4, HXT7, MEP3, PHS1, SEC59, SSS1, TPO1, VMA16*, and *YPQ1*. Interaction effects were heterogeneous in direction: Several *HXT* events increased under stress in WT relative to 4KR, whereas several *MEP3* events showed the opposite pattern. The interaction demonstrates a difference in stress-associated local occupancy between genotypes; it does not establish direct action of eS7 ubiquitination on each substrate.

**Figure 6.**
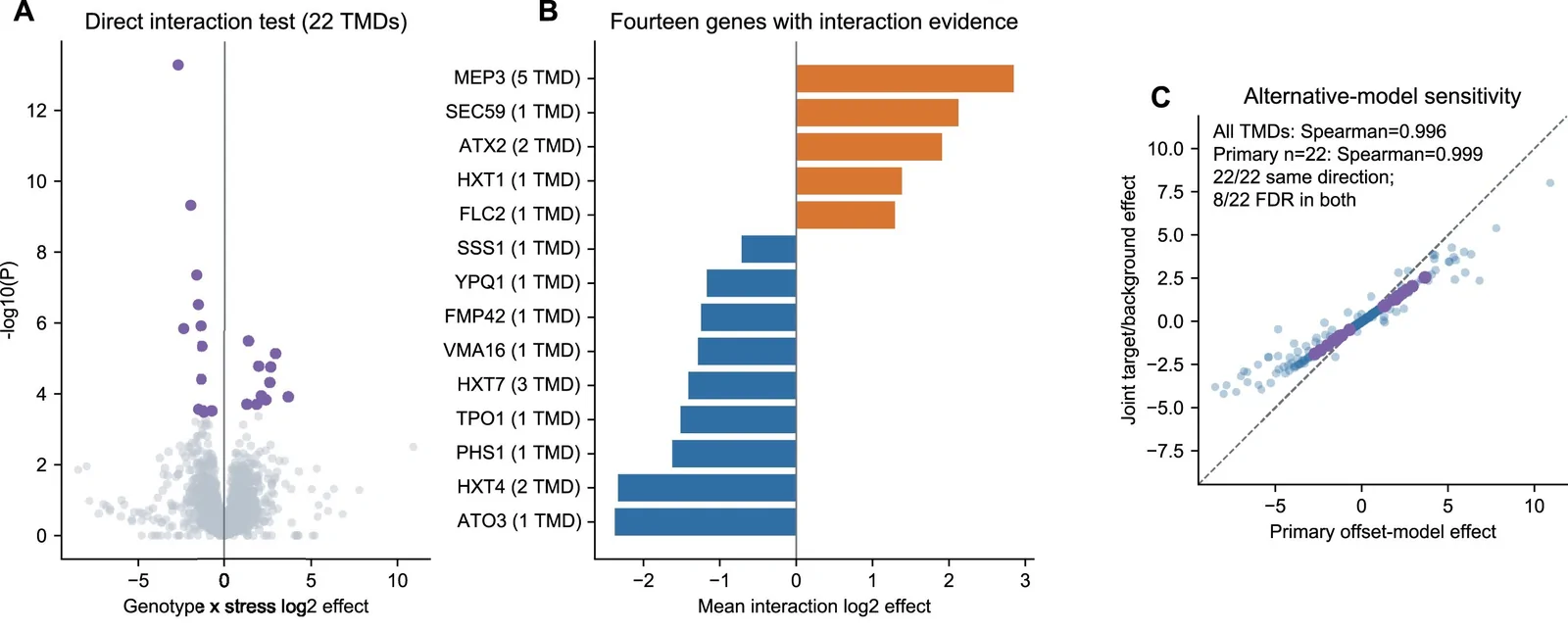
Direct genotype-by-stress modeling identifies eS7-associated differences. (**A**) Volcano plot for the interaction; points with FDR < 0.05 are highlighted. (**B**) Gene-level summaries for 22 interaction-positive TMDs in 14 genes; parenthetical labels give the number of TMDs per gene. (**C**) Agreement between the primary offset model and the alternative joint target/background sensitivity model, reported across all tested TMDs and within the 22-TMD primary set. All 22 primary interaction TMDs retained their effect direction, but only eight met FDR < 0.05 in both models. The dashed line indicates identity.

The alternative target/background sensitivity model produced concordant primary effect estimates (Spearman ρ = 0.996; 99.2% direction agreement across 2892 TMDs). Among the 22 primary interactions specifically, effect estimates had Spearman ρ = 0.999 and Pearson r = 1.000, all 22 retained their direction, and eight met the FDR < 0.05 criterion in both models (Fig. 6C and Supplementary Fig. S5). Nine interactions met the FDR < 0.05 criterion in the sensitivity model overall. These comparisons assess robustness to background treatment and do not constitute independent confirmation of 22 events. The common-offset analysis provided a separate calibration check: among all 2892 TMDs testable under both calibrations, interaction effects had Spearman ρ = 0.992 with the sample-specific-offset model; among the 22 primary interactions, Spearman ρ = 0.999, all directions were retained, and 21 had FDR < 0.05 in the common-offset model. Those common-offset FDRs were Benjamini–Hochberg adjusted across all 2895 TMDs testable in that model (Supplementary Fig. S8 and Supplementary Table S4). Across the 11-position scan, 22 interactions were significant at +75 nt under center-wise FDR and 16 remained significant under global correction across the complete family of 32 723 tested TMD-by-position hypotheses (Supplementary Fig. S3). All 22 primary interaction TMDs retained their effect direction in the joint target/background model, but only 8/22 met FDR < 0.05 in both models. The remaining 14 are primary-model findings with model-dependent statistical support. Common-offset calibration and position-wide correction address separate sensitivity questions.

The 22 interaction-positive TMDs should not be interpreted as 22 independent biological events because several arise from the same transcript and share gene-level background information. They map to 14 genes; the number of interaction-positive TMDs and the strongest TMD-level effect for every gene are therefore reported as a descriptive gene-level summary in Supplementary Table S4.

A dedicated analysis of the response-associated subsets identified 41 WT stress-responsive TMDs in 29 genes, six 4KR stress-responsive TMDs in six genes, and 22 genotype-by-stress interaction TMDs in 14 genes. At the protein level, single-pass proteins represented 2/29, 0/6, and 1/14 selected genes, respectively, compared with 31.5%–32.2% of nonselected genes. Although the unadjusted WT comparison suggested single-pass depletion (Fisher *P* = .00334), this association did not persist after exact conditioning on tested-TMD opportunity (*P* = .421); the 4KR and interaction exact *P-*values were 1.0, and all topology FDRs were 1.0. None of the 18 prespecified sequence-feature tests passed global FDR correction. The strongest nominal association was GRAVY in the interaction set (log-odds coefficient = 0.641 per SD, *P* = .0106, global FDR = 0.190). Results for the six-event 4KR set were considered descriptive. Thus, no robust common topology class or association with the tested sequence features was detected for the response-associated sets (Supplementary Table S4).

### Functional enrichment is sensitive to per-gene testing opportunity

The 22 interaction TMDs mapped to 14 genes within a universe of 772 testable membrane-protein genes. Using the analyzable universe alone, broad transmembrane transporter activity and transmembrane transport were enriched (Fisher FDR = 0.0173 for both). After accounting for the number of tested TMDs per gene, however, these broad terms were no longer significant (opportunity-adjusted FDR = 0.282 and 0.426, respectively; Fig. 7 and Supplementary Fig. S7).

**Figure 7.**
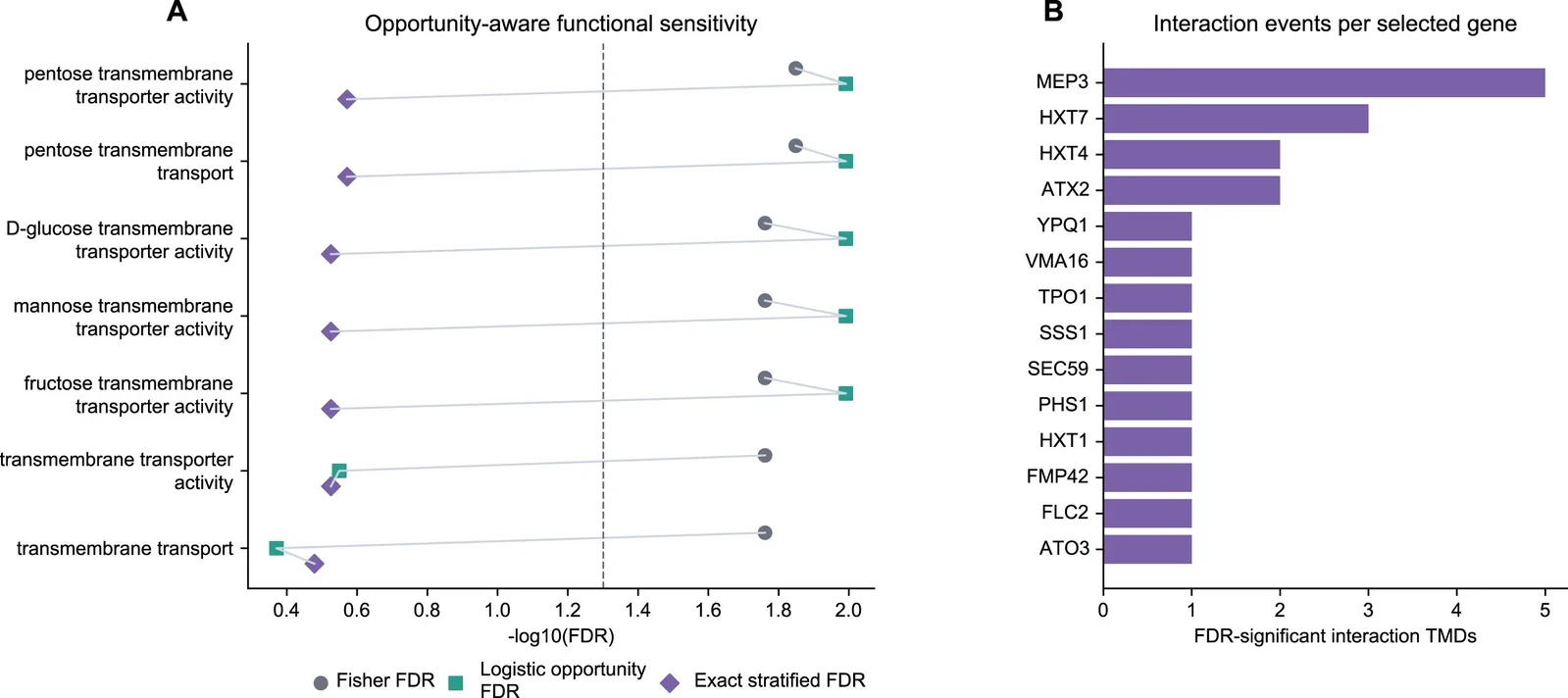
Functional conclusions depend on per-gene testing opportunity. (**A**) Comparison of opportunity-unadjusted Fisher FDRs, asymptotic logistic FDRs adjusted for the number of tested TMDs per gene, and exact FDRs conditioned on the same tested-TMD-count strata for selected broad and narrow GO terms. The vertical line marks FDR 0.05. (**B**) Numbers of FDR-significant interaction TMDs contributed by the 14 interaction genes. Per-gene numbers of tested TMDs are shown in Supplementary Fig. S7B.

Several narrower sugar-transport categories were significant in the asymptotic logistic analysis and were driven by *HXT1, HXT4*, and *HXT7*. However, none of the 1502 tested terms passed FDR correction in the exact opportunity-stratified analysis; for the leading HXT-linked terms, nominal exact *P-*values ranged from 3.57 × 10^−4^ to 0.0040 but FDRs ranged from 0.268 to 0.298 (Fig. 7 and Supplementary Fig. S7 and Supplementary Table S5). Functional enrichment is therefore presented only as an exploratory description of this small 14-gene set, not as evidence for a general transporter or HXT-specific regulatory program.

## Discussion

### The principal signal is downstream of the TMD boundary

The spatial scan changes the interpretation of a boundary-centered score. The strongest reproducible baseline signal occurs around +75 nt, not at the annotated TMD start. At +75 nt, the ribosomal P site lies approximately 25 codons downstream of the annotated TMD start, placing the nascent TMD near the stage at which its N-terminal portion may approach or begin to emerge from the ribosomal exit tunnel. The signal is therefore compatible with processes associated with cotranslational emergence, targeting, translocon engagement, insertion, or another downstream event [2–5], but ribosome footprints alone cannot distinguish these mechanisms or prove a discrete elongation pause.

Recent selective ribosome-profiling experiments in yeast showed that SRP preferentially engages TMDs as they emerge from the ribosomal tunnel and that SRP-bound substrates exhibit increased collided-ribosome footprints. Conventional monosome profiles, however, did not show comparable accumulation at SRP-binding onset [21]. These findings provide relevant biological context but do not identify the origin of the +75-nt monosome signal analyzed here. The present study instead tests how TMD-centered local ribosome occupancy changes with ER stress and eS7 genotype.

Equal weighting of normalized TMD profiles prevents highly expressed transcripts from dominating the metagene average, but multi-pass proteins can still contribute more than one TMD profile. Protein-level summaries and gene-clustered analyses were used for topology comparisons, with one-TMD-per-protein resampling additionally assessing sensitivity to unequal TMD averaging. Gene-wide non-TMD background further reduces transcript-abundance effects while avoiding neighboring-TMD and CDS-boundary contamination. Together, these changes provide a more interpretable measure of spatially resolved local accumulation.

### Sequence associations and sensitivity of topology comparisons

The baseline distribution argues against a universal TMD-associated ribosome signal. Aromatic-residue fraction was the only configured sequence term that remained significant after FDR correction in the joint model. Aromatic residues may influence nascent-chain conformation, recognition, topology, or insertion energetics, but the observational association does not identify a mechanism. Other marginal composition and flank-charge associations are best treated as candidates for future tests.

Equal protein weighting does not equalize the number of TMDs contributing to each protein mean. The original mean-based upper-tail odds ratios attenuated substantially under one-TMD-per-protein sampling, while the continuous protein-level and gene-clustered TMD-level comparisons were nonsignificant. The mean-based association is therefore no longer interpreted as evidence for biological enrichment of single-pass proteins in the high-signal tail.

### Replicate-aware inference narrows and clarifies stress responses

The count-level models identify 41 WT and six 4KR stress-responsive TMDs after multiple-testing correction. Within-condition replicate correlations were moderate rather than perfect, emphasizing why significance cannot be inferred from a large pooled change alone. The quasi-likelihood framework incorporates biological variation directly.

The overlap analysis shows that baseline high signal, within-genotype stress response, and genotype-by-stress interaction are related but not interchangeable. Basal high signal overlapped all three TMD-level response sets after restriction to the 2208-TMD baseline-eligible intersection, but the absolute overlap counts were only seven, two, and six, and the 4KR comparison contained only three eligible responsive TMDs. The strong overlap between WT stress effects and interactions in the full primary universe is expected when a stress response is pronounced in WT and attenuated or reversed in 4KR. These overlaps therefore support a relationship among the signal classes without making baseline accumulation a sufficient predictor of stress response.

Dedicated response-set characterization further separates correlates of baseline accumulation from correlates of differential response. Neither the tested sequence features nor the opportunity-adjusted topology comparisons were FDR significant for the WT stress, 4KR stress, or genotype-by-stress interaction sets. The nominal single-pass depletion in the WT response set did not persist after conditioning on tested-TMD opportunity. Given this result and the aggregation sensitivity of the baseline protein-mean tail association, their descriptive contrast should not be interpreted as a biological reversal. The overall response-set analysis does not establish an absence of biological structure, particularly for the six-event 4KR set, but it argues against attributing the differential sets to the baseline correlates alone. Functional and localization patterns therefore remain exploratory hypotheses rather than a unified sequence- or topology-defined response class.

### Primary interaction findings and their sensitivity to background modeling

Coordinate-consistent, replicate-aware analysis identified direct genotype-by-stress interactions at 22 TMDs in 14 genes. These results are based on direct interaction testing rather than separate threshold calls in WT and 4KR.

The alternative region sensitivity model propagates target/background contrast uncertainty and produces nearly identical effect ranks and directions, including among the 22 primary interaction-positive TMDs, although only eight of the 22 met the FDR < 0.05 criterion in both models. A common-offset analysis likewise retains the direction of all 22 primary interactions, with 21 meeting the FDR < 0.05 criterion after correction across the common-offset model’s complete 2895-TMD test universe, arguing against genotype-correlated offset calibration as their source. The global positional analysis also retains 16 +75-nt interactions after correction over the 32 723-test scan family. Directional concordance therefore extends to all 22 primary findings, whereas shared FDR support is restricted to 8/22. The remaining 14 are primary-model findings with model-dependent statistical support. The common-offset and position-wide analyses address separate sensitivity questions and do not provide independent confirmation. They do not establish whether the differences are direct consequences of eS7 ubiquitination or indirect effects of altered stress adaptation.

### Functional interpretation requires opportunity correction

Restricting GO analysis to analyzable membrane-protein genes prevents enrichment of membrane categories by construction. An additional correction is necessary because a gene with many tested TMDs has more opportunities to contain an interaction. The exact opportunity-stratified analysis removes FDR-level support for both broad transporter terms and narrower HXT-linked sugar-transport categories. The nominal concentration of *HXT1, HXT4*, and *HXT7* remains biologically interesting, but with only 14 selected genes it is hypothesis generating rather than confirmatory.

### Limitations and future directions

This study reuses one published dataset and inherits its experimental design. Independent Ribo-seq data and orthogonal assays are required to establish generality and determine whether local footprint signals reflect elongation time, ribosome collisions, targeting, insertion, or another process. Matched RNA-seq data are available in the source study. The present target-versus-within-gene-background analysis was designed to quantify spatial redistribution of ribosome occupancy rather than transcript-level abundance; explicit integration of the matched RNA-seq data would nevertheless help separate transcript-abundance changes from local translational redistribution.

Coverage and structural eligibility vary by sample and position. The strict eight-sample intersection was used for primary inference, and every population is reported explicitly. The alternative target/background sensitivity model addresses one important source of uncertainty, but it does not remove all count-model assumptions. Sequence and GO results are associative, and the small number of selected genes limits mechanistic interpretation.

The reusable workflow improves reproducibility and permits application to other datasets, but it is not a universally validated pausing detector. External users must validate organism-specific annotations, P-site calibration, biological replication, and model design. Targeted reporter experiments at representative *HXT, MEP3*, and other interaction TMDs would provide the clearest next test.

## Supplementary Material

lqag137_Supplemental_Files

## Data Availability

The ribosome-profiling datasets reanalyzed in this study are publicly available from the Gene Expression Omnibus under accession GSE128578. No new sequencing data were generated. Core processed data, quality-control summaries, model outputs, configuration files, and figure source tables are provided as Supplementary Tables and in the public repository at https://github.com/daming7977-byte/TMD-RiboSeq (tmdriboseq v1.0.0 core; paper-reproducibility commit 2dc3003456fd9b6f704b8c6fdc584c78ada4abac). The repository contains configs/paper_v1.0.yaml for paper reproduction and documented instructions for external BAM, annotation, TMD, and sample-metadata inputs. The same 120-file core source tree is supplied as Supplementary Source Code v1.0.0. The additional one-TMD-per-protein scripts, fixed parameters, frozen inputs, and resampling outputs are supplied separately as the Supplementary Source Code R2 Addendum, with upper-tail summaries in Supplementary Table S3.
