# Supplementary material for: Genome-wide analysis of TMD-associated local ribosome accumulation reveals stress- and eS7-associated remodeling in Saccharomyces cerevisiae
Source: NAR Genom Bioinform. 2026 Nov 10;8(4):lqag137. doi: 10.1093/nargab/lqag137 (PMC13748878; doi:10.1093/nargab/lqag137)
Supplement: Supplementary file 1 [file lqag137_supplemental_files.zip › Supplementary_Figures_S1-S8_R2.pdf]

Supplementary Figures S1–S8

NARGAB-2026-201

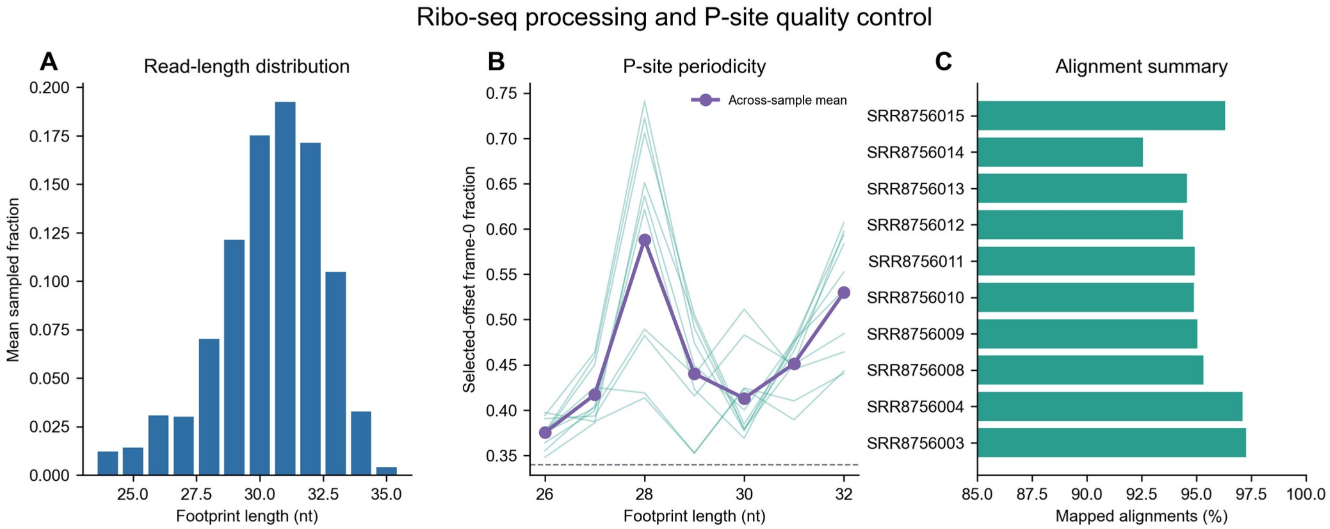

Supplementary Figure S1. Ribo-seq processing and P-site quality control.

(A) Across-sample footprint-length distribution. (B) Selected-offset frame-0 fractions by footprint length. (C) Mapping percentages.

Alt text: Three panels show canonical footprint lengths, read-length-dependent coding-frame periodicity, and high alignment percentages for ten Ribo-seq samples.

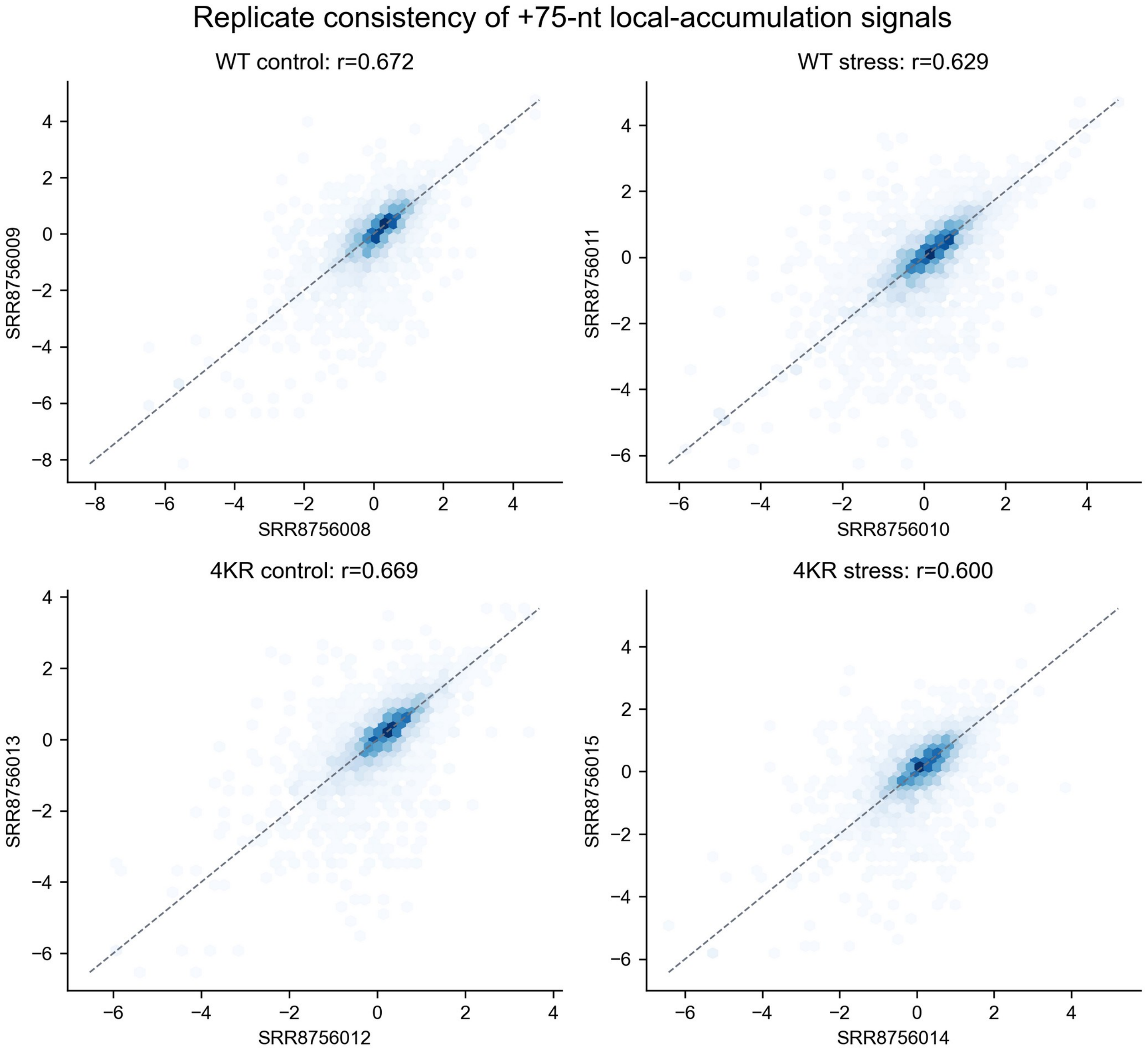

Supplementary Figure S2. Replicate consistency of +75-nt local-accumulation signals.

Hexbin plots compare biological replicates using TMDs that passed coverage in both samples.

Alt text: Four dense diagonal plots show moderate replicate agreement in WT control, WT stress, 4KR control, and 4KR stress cells, with Pearson correlations from 0.600 to 0.672.

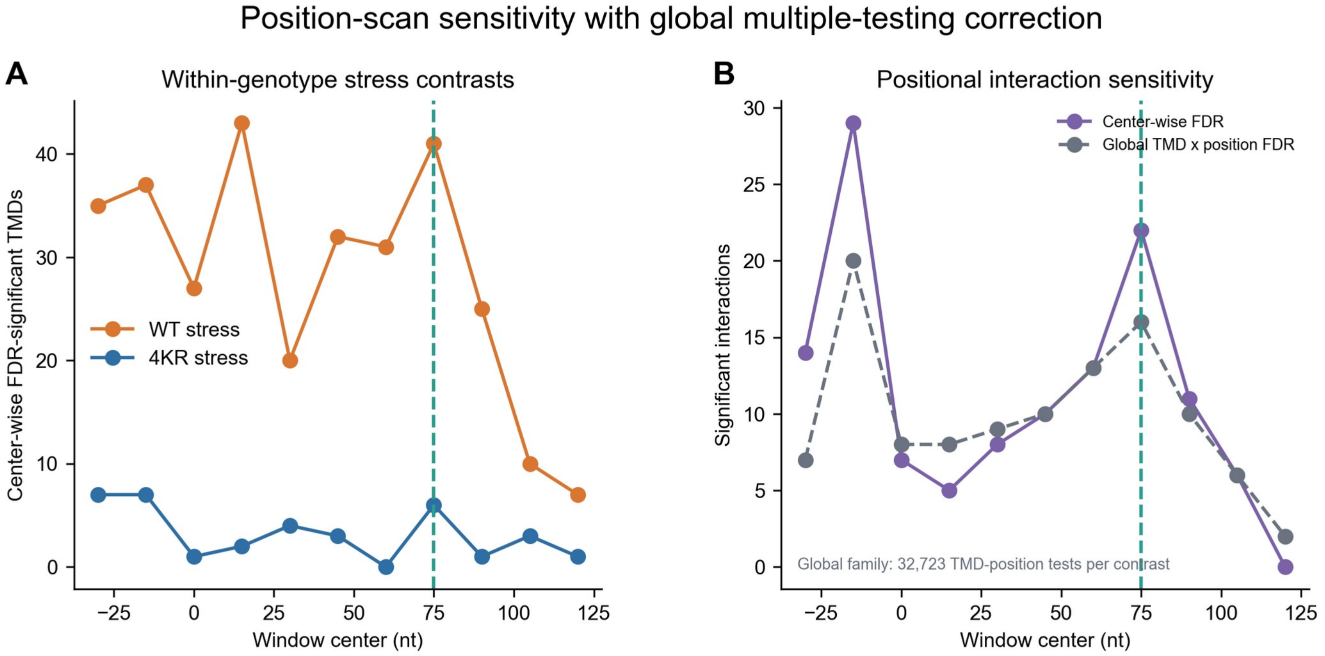

Supplementary Figure S3. Positional sensitivity with global multiple-testing correction.

(A) Numbers of center-wise FDR-significant WT and 4KR stress effects across 11 positions. (B) Center-wise and global TMD-by-position significant interactions. Global FDR was calculated over 32,723 tested TMD-by-position hypotheses per contrast. The dashed line marks +75 nt.

Alt text: Two line plots summarize 11 scanned positions and a 32,723-hypothesis global correction family, showing that 16 interactions remain globally significant at the +75-nt primary position.

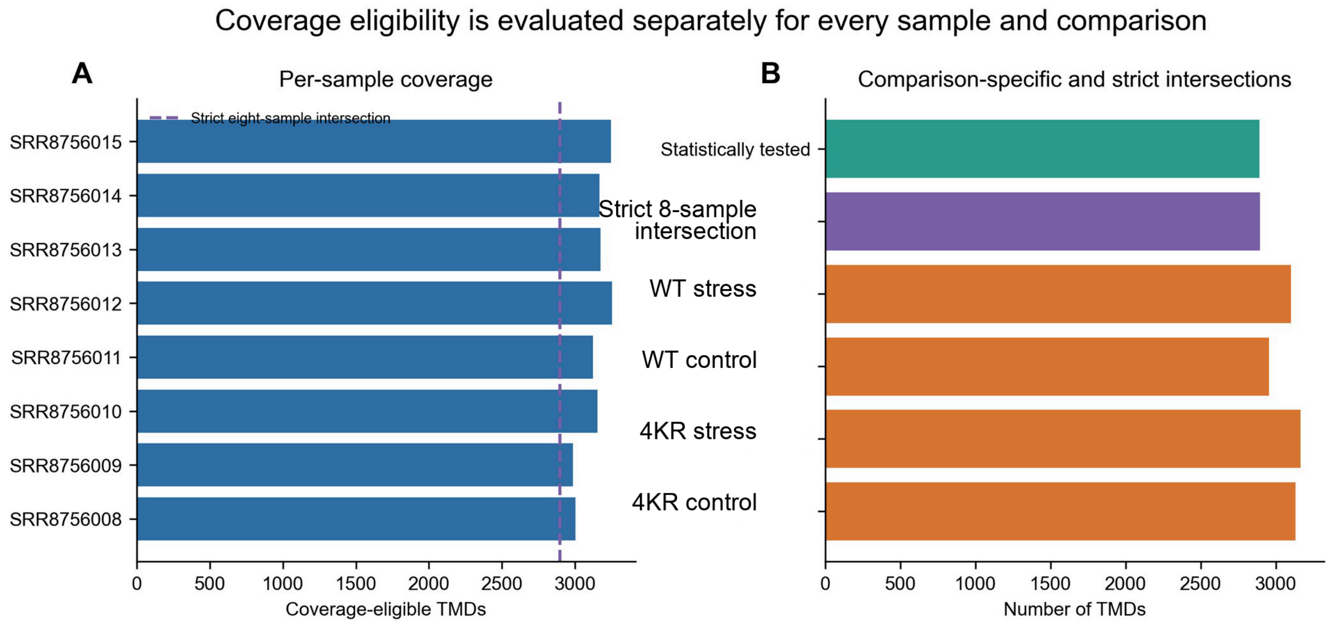

Supplementary Figure S4. Coverage eligibility by sample and comparison.

(A) Per-sample eligible TMD counts and the strict eight-sample intersection. (B) Design-cell, strict-intersection, and statistically tested populations.

Alt text: Horizontal bars show coverage-eligible counts for each sample and comparison, including 2,896 TMDs in the strict intersection and 2,892 statistically tested TMDs.

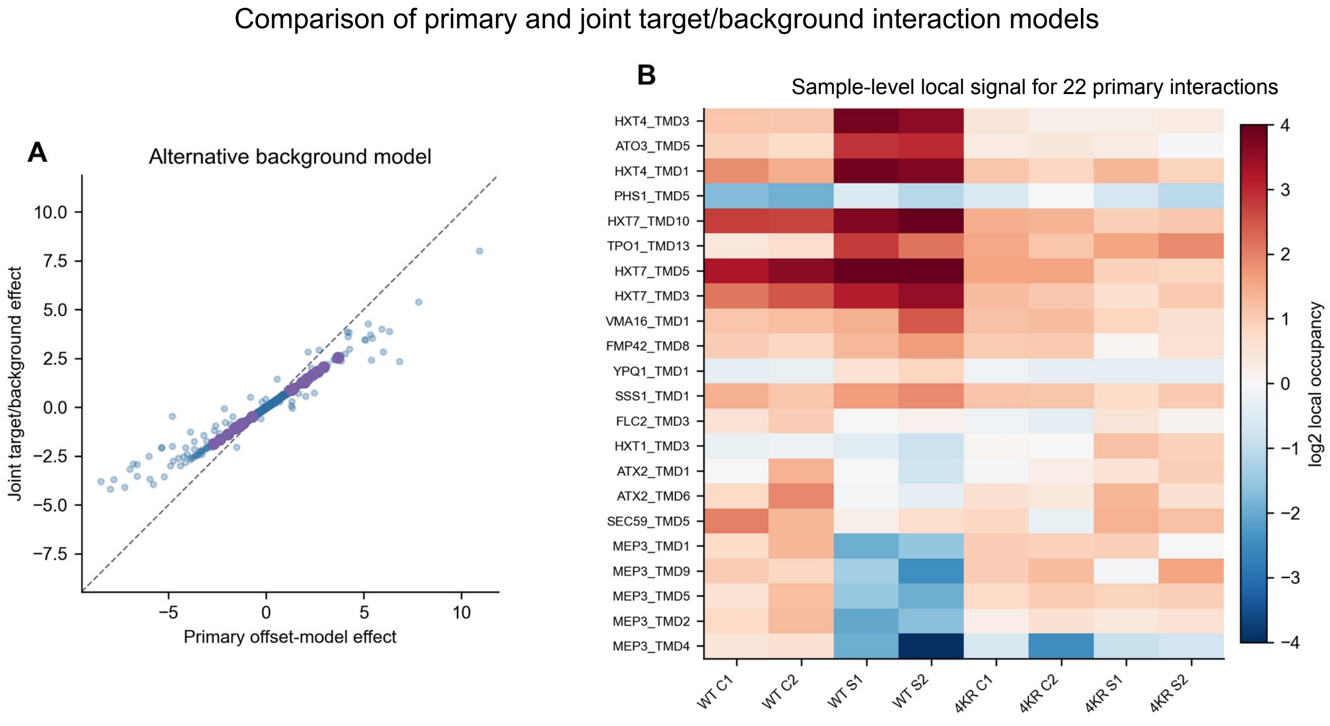

Supplementary Figure S5. Comparison of primary and joint target/background interaction models.

(A) Interaction effects from the primary and joint target/background models; purple points denote the 22 primary FDR-significant TMDs. All 22 retained their effect direction, but only eight met FDR <0.05 in both models. The dashed line indicates identity. (B) Sample-level local signals for the 22 primary interaction TMDs.

Alt text: An effect-comparison plot shows concordant directions for all 22 primary interaction TMDs, with eight meeting FDR <0.05 in both models. A 22-row heat map shows their sample-level genotype-stress patterns.

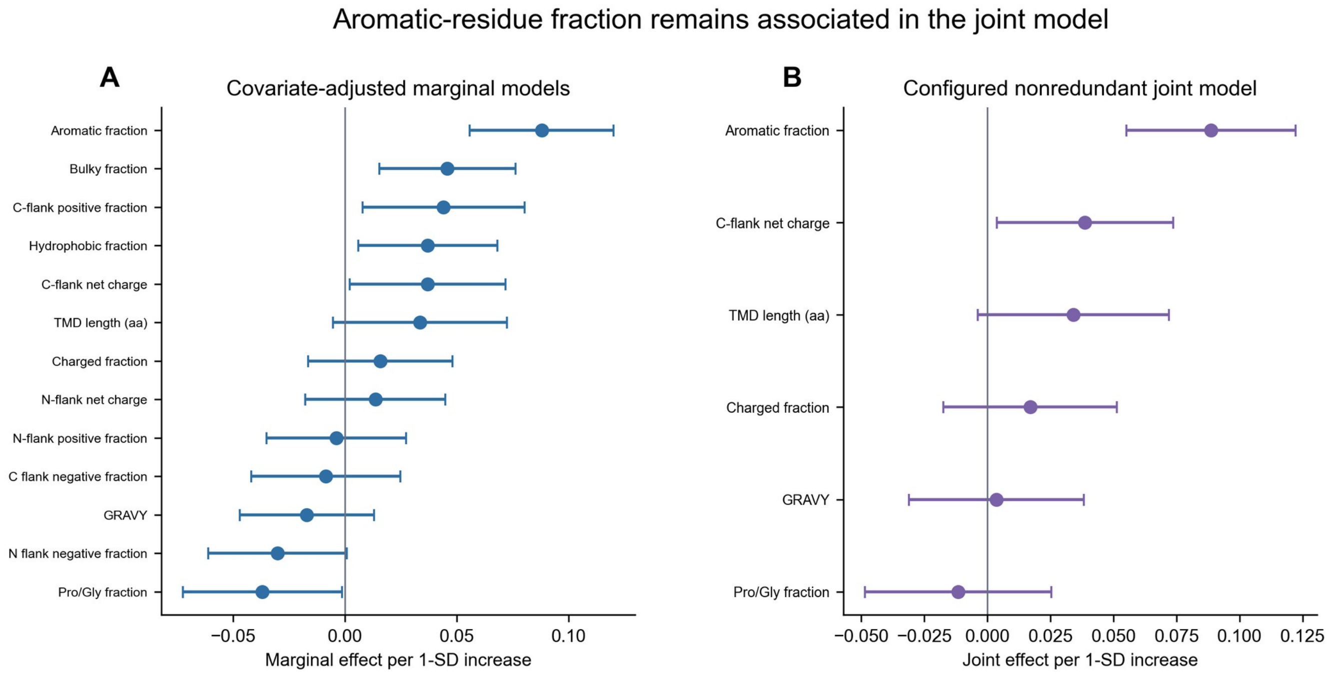

Supplementary Figure S6. Sequence-feature associations with baseline local accumulation.

(A) Covariate-adjusted marginal coefficients. (B) Configured nonredundant joint-model coefficients. Error bars show 95% confidence intervals.

Alt text: Two forest plots show several marginal composition associations but only aromatic-residue fraction remaining FDR significant in the joint model.

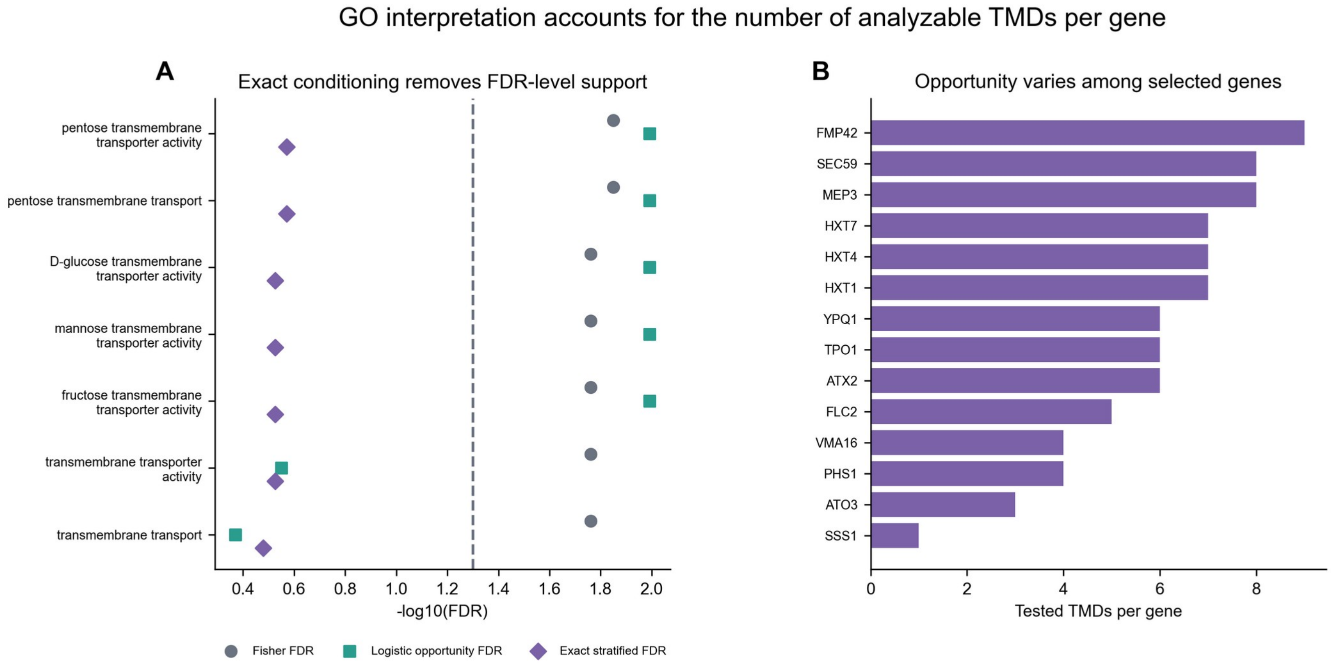

Supplementary Figure S7. GO interpretation accounts for tested TMD opportunity.

(A) Fisher, asymptotic logistic opportunity-adjusted, and exact opportunity-stratified FDRs for representative GO terms. (B) Tested TMD counts for the 14 interaction genes.

Alt text: Exact opportunity-stratified testing removes FDR-level support for both broad and narrow transport terms; selected genes have between one and nine tested TMDs.

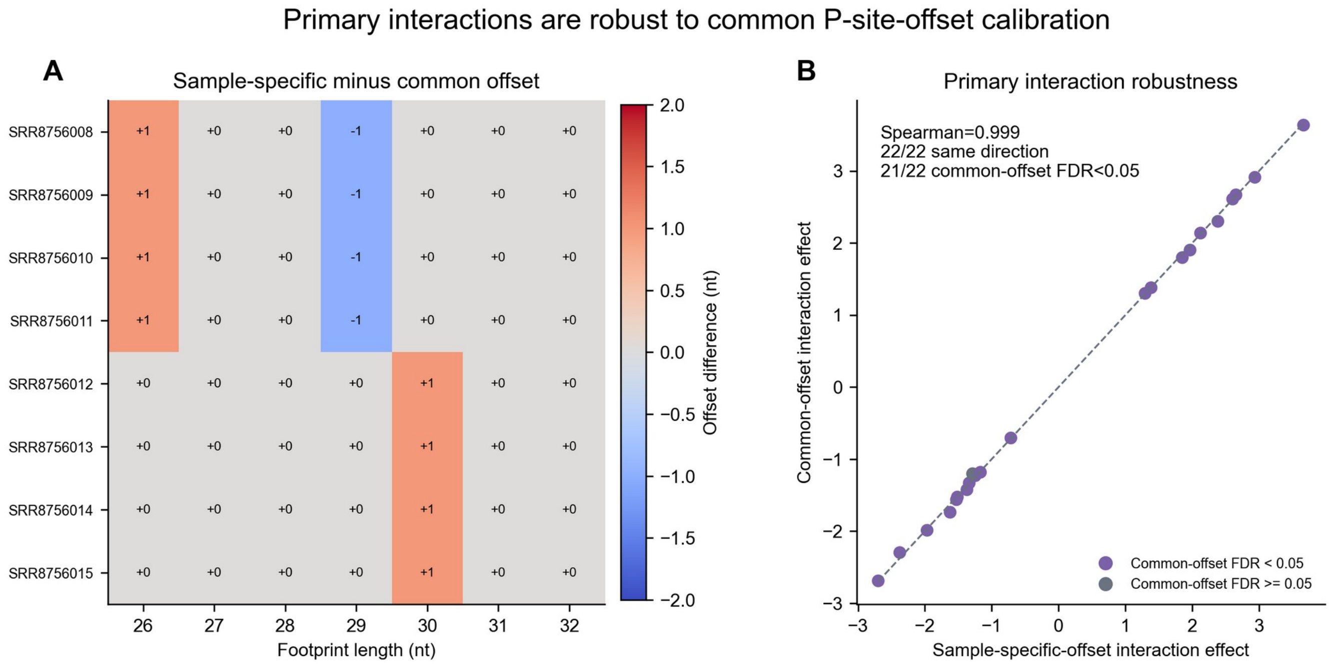

Supplementary Figure S8. Primary interaction effects are robust to a common P-site-offset calibration.

(A) Differences between sample-specific and common read-length-specific offsets for the eight modeled libraries. Common offsets were selected from coding-frame periodicity only. (B) Interaction effects for the 22 primary TMDs under sample-specific and common-offset calibration; purple points have common-offset FDR <0.05 and gray points have common-offset FDR >=0.05.

Alt text: An offset audit shows the sample-by-read-length assignments changed by consensus calibration, while a near-diagonal scatter plot shows that all 22 primary interaction effects retain direction and 21 have FDR below 0.05 after correction across all 2,895 TMDs tested by the common-offset model.
