# Supplementary material for: Genome-wide analysis of TMD-associated local ribosome accumulation reveals stress- and eS7-associated remodeling in Saccharomyces cerevisiae
Source: NAR Genom Bioinform. 2026 Nov 10;8(4):lqag137. doi: 10.1093/nargab/lqag137 (PMC13748878; doi:10.1093/nargab/lqag137)
Supplement: Supplementary file 1 [file lqag137_supplemental_files.zip › Supplementary_Source_Code_R2_Addendum.zip!/TMD-RiboSeq-R2-Addendum/r2_addendum/figures/Figure_4D.pdf]

D Sensitivity to the per-protein TMD sampling scheme

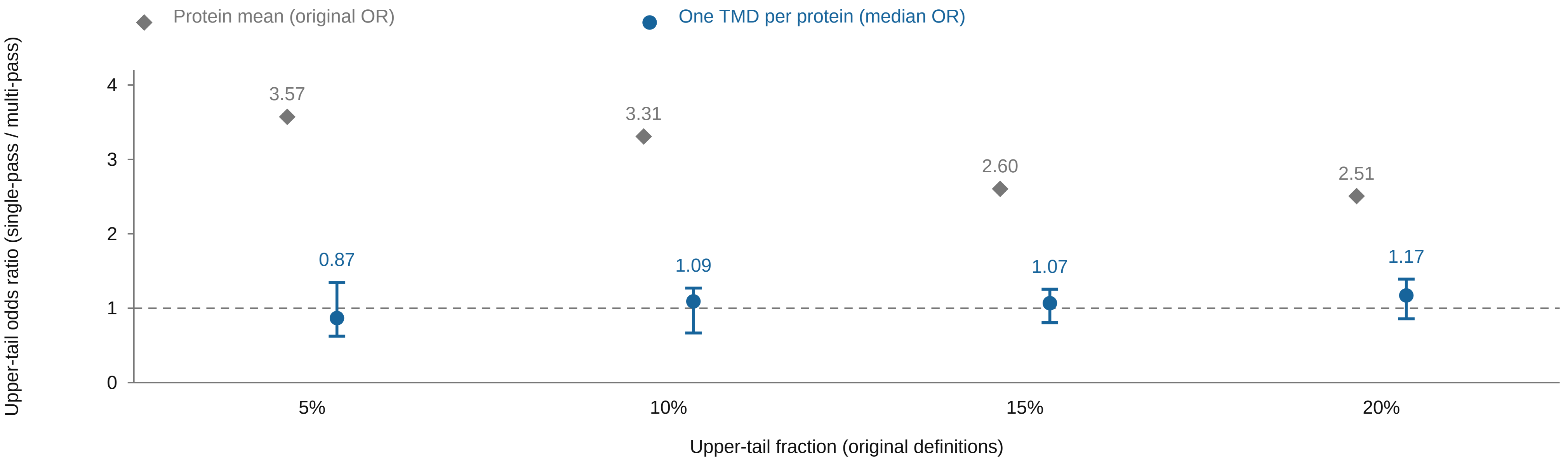

Blue bars: 2.5–97.5% resampling ranges, not confidence intervals. 10,000 iterations; 615 fixed proteins.
